# The Pentameric Nucleoplasmin Fold Is Present in *Drosophila* FKBP39 and a Large Number of Chromatin-Related Proteins

**DOI:** 10.1016/j.jmb.2015.03.010

**Published:** 2015-05-22

**Authors:** Christian Edlich-Muth, Jean-Baptiste Artero, Phil Callow, Marcin R. Przewloka, Aleksandra A. Watson, Wei Zhang, David M. Glover, Janusz Debski, Michal Dadlez, Adam R. Round, V. Trevor Forsyth, Ernest D. Laue

**Affiliations:** 1 Department of Biochemistry, University of Cambridge, 80 Tennis Court Road, CB2 1GA Cambridge, United Kingdom; 2 Life Sciences Group, Institut Laue-Langevin, 71 Avenue des Martyrs, CS 20156, Grenoble, Cedex 9, France; 3 Faculty of Natural Sciences, Keele University, ST5 5BG Staffordshire, United Kingdom; 4 Department of Genetics, University of Cambridge, Downing Street, CB2 3EH Cambridge, United Kingdom; 5 Mass Spectrometry Laboratory, Department of Biophysics, Institute of Biochemistry and Biophysics, Polish Academy of Sciences, 5A Pawinskiego Street, 02-106 Warsaw, Poland; 6 European Molecular Biology Laboratory, Grenoble Outstation, 71 Avenue des Martyrs, 38042 Grenoble, France; 7 Unit for Virus Host–Cell Interactions, University Grenoble Alpes–European Molecular Biology Laboratory–CNRS, 71 Avenue des Martyrs, 38042 Grenoble, France

**Keywords:** SANS, small-angle neutron scattering, SAXS, small-angle X-ray scattering, NOE, nuclear Overhauser enhancement, HSQC, heteronuclear single quantum coherence, PPI, peptidyl proline isomerase, histone chaperone, nucleoplasmin, FKBP, structure determination, NMR

## Abstract

Nucleoplasmin is a histone chaperone that consists of a pentameric N-terminal domain and an unstructured C-terminal tail. The pentameric core domain, a doughnut-like structure with a central pore, is only found in the nucleoplasmin family. Here, we report the first structure of a nucleoplasmin-like domain (NPL) from the unrelated *Drosophila* protein, FKBP39, and we present evidence that this protein associates with chromatin. Furthermore, we show that two other chromatin proteins, *Arabidopsis thaliana* histone deacetylase type 2 (HD2) and *Saccharomyces cerevisiae* Fpr4, share the NPL fold and form pentamers, or a dimer of pentamers in the case of HD2. Thus, we propose a new family of proteins that share the pentameric nucleoplasmin-like NPL domain and are found in protists, fungi, plants and animals.

## Highlights

• The FKBP39 N-terminal domain has the nucleoplasmin fold and forms a homo-pentamer.
• FKBP39 binds to chromatin, interacts with histones and binds divalent metal ions.
• Other proteins such as the yeast Fpr4 and plant HD-tuins are expected to have the same N-terminal domain with similar properties.

## Graphical abstract

**Figure:**
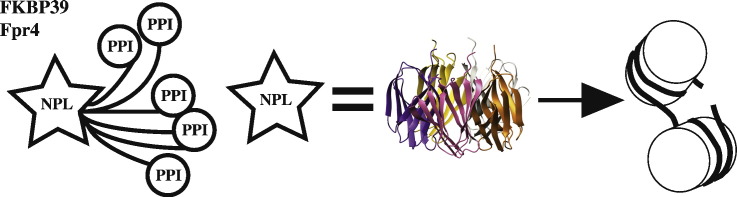

## Introduction

Histone chaperones sequester histones. They either temporarily bind and present histones to histone modifying enzymes or hold histones in an inactive state after they are synthesised or released from DNA during transcription, replication and repair. In particular, histone chaperones prevent potentially toxic histone proteins from binding DNA, a role that is exemplified by the amphibian nucleoplasmin protein. Nucleoplasmin (NUP) provides a long-lasting store of histones H2A/H2B in *Xenopus* eggs, which is sufficient for a dozen or so rounds of DNA replication after fertilisation. It is also involved in the de-condensation of sperm chromatin (reviewed in Ref. [1]). Nucleoplasmin's many phosphorylated residues, as well as some acidic loops, seem to play a part in binding positively charged histones. Indeed, poly-glutamate (mimicking the C-terminal tail of nucleoplasmin) has also been found to de-condense sperm chromatin, presumably through binding positively charged histones, albeit less efficiently than nucleoplasmin [2–4]. The highly thermostable N-terminal domain of NUP alone is also capable of binding histones. Its structure was solved [5] revealing a homo-pentamer, prompting the question of how a molecule with 5-fold symmetry might interact with histone complexes, which have even-numbered symmetry. An electron microscopy study of native hyper-phosphorylated NUP concluded that five H2A/H2B dimers bind one NUP core particle or one histone dimer per NUP subunit [6].

Perhaps due to its unique role, nucleoplasmin's similarity to a group of proteins implicated in chromatin regulation, such as the plant type 2 histone deacetylases, or HD-tuins [7–11], and the yeast Fpr3 and Fpr4 proteins [12–14], has never been noticed. The family's defining feature is an N-terminal domain of unknown structure with a signature motif of conserved hydrophobic and two potentially catalytic residues—a histidine and an aspartate [15]. Nucleoplasmin has never been included in this group because it lacks these “catalytic” residues and is also, in other respects, a very distant relative to the extent that standard search engines will not retrieve any nucleoplasmins when queried with, for example, Fpr4. Nonetheless, we show that all of these proteins are likely to have a nucleoplasmin-like domain (NPL) at their N-termini with the same three-dimensional structure.

## Results

### Phylogenetic overview

**Fig. 1:**
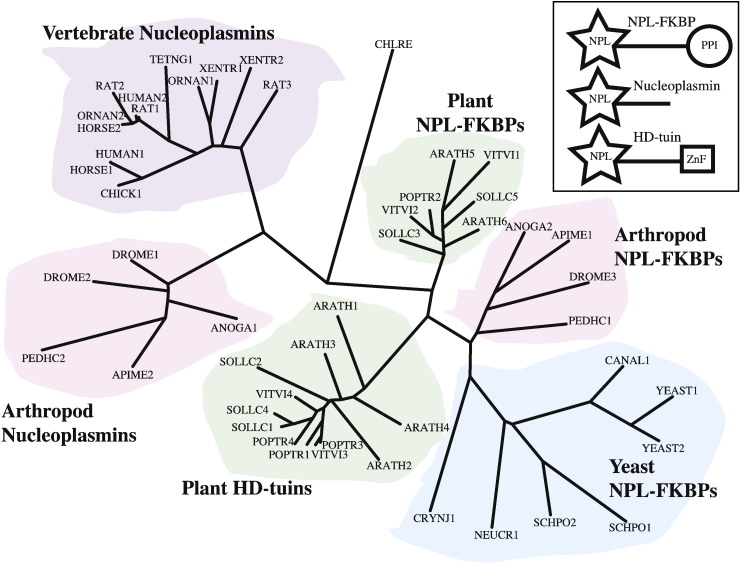
Phylogeny of the nucleoplasmin-like domain. NPL proteins were selected from representative vertebrates, arthropods, plants and yeast species. Protozoa are omitted except for one representative, *Chlamydomonas reinhardtii* (CHLRE). For each species, all NPL proteins are included and labelled with a species tag and a number starting with one. *Drosophila* FKBP39 is DROME3, *S*. *cerevisiae* Fpr4 is YEAST1 and *Arabidopsis* HD2a is ARATH3. Abbreviated species are ANOGA: *Anopheles gambiae*, APIME: *A*. *mellifera*, ARATH: *A*. *thaliana*, CANAL: *Candida albicans*, CRYNJ: *Cryptococcus neoformans*, DROME: *D*. *melanogaster*, NEUCR: *Neurospora crassa*, ORNAN: *Ornithorhynchus anatinus*, PEDHC: *Pediculus humanus*, POPTR: *Populus trichocarpa*, SCHPO: *Schizosaccharomyces pombe*, SOLLC: *Solanum lycopersicum*, TETNG: *Tetraodon nigroviridis*, VITVI: *Vitis vinifera* and XENTR: *Xenopus tropicalis*. The full names of species and proteins are listed in Supplementary Table 1. Inset: NPL proteins can be classified as NPL-FKBPs, HD-tuins or nucleoplasmins, based on the presence or absence of a C-terminal domain, either a PPI or a C_2_H_2_ zinc finger (ZnF). Nucleoplasmins have no C-terminal domain, only an unstructured “tail”. The NPL domain is always at the N-terminus.

NPL proteins exist in three different multi-domain arrangements (Fig. 1, inset). NPL-FKBPs possess a C-terminal peptidyl proline isomerase (PPI) domain and plant HD-tuins have a C-terminal zinc finger. Nucleoplasmins do not contain an additional domain. Following the N-terminal NPL, all of these proteins feature long stretches (more than 100 residues) of sequence that are highly charged, predominantly acidic and likely unstructured.

**Fig. 2:**
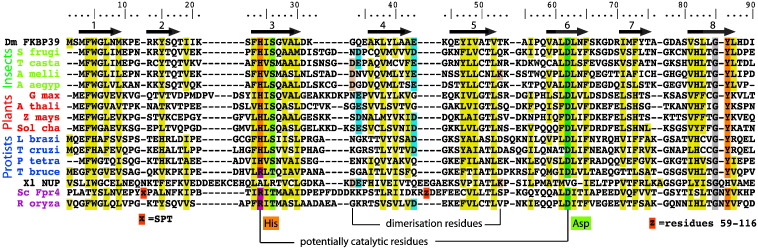
Structural alignment of NPL sequences. The structures of *Xenopus* nucleoplasmin (1KJ5, labelled “Xl NUP”) and FKBP39 were aligned and the other sequences were added based on sequence only. The two other proteins used in our experiments are yeast Fpr4 and *A*. *thaliana* HD2 (“A thali”). The residue numbering and the position of β-strands of FKBP39 are indicated above its sequence. The positions of the conserved and potentially catalytic histidine and aspartate are indicated, as well as the two dimer-promoting residues of nucleoplasmin. The yeast protein Fpr4, also an NPL-FKBP, contains a large insertion (residues 59–116, indicated by “z”) that has been omitted from the alignment. The other proteins are from the fungus *Rhizopus oryzae*; the insects *Spodoptera frugiperda*, *Tribolium castaneum*, *A*. *mellifera* and *Aedes aegypti*; the plants *Glycine max*, *A*. *thaliana*, *Zea mays* and *Solanum chacoense*; and the protozoa *Leishmania braziliensis*, *Trypanosoma cruzi*, *Paramecium tetraurelia* and *Trypanosoma brucei*.

The phylogenetic tree of NPL sequences from plants, fungi and animals (Fig. 1) clearly shows that NPL-FKBPs and HD-tuins form their own well-separated clades. However, the major divide is between nucleoplasmins and the other two groups, with as little as 12% pairwise sequence identity between them. This can also be observed in the sequence alignment (Fig. 2) where only the hydrophobic character of some residues and the position of the β-strands are preserved between *Xenopus* nucleoplasmin (third to the last sequence in the alignment) and all the other proteins. In order to investigate whether all of these sequences share a common three-dimensional fold, we set out to determine the structure of a non-nucleoplasmin NPL.

### Structure determination of the N-terminal domain of FKBP39

The *Drosophila* FKBP39 protein (also known as FK506-binding protein 1, or FK506-bp1) falls into the arthropod subgroup of proteins that have a PPI domain at the C-terminus. FKBP39 was chosen for structural studies because its NPL domain does not contain lengthy loops or insertions but does contain the putative catalytic residues. Residues 1–92 were expressed as a GB1 (protein G, B1 domain) fusion protein and were found to form a highly thermostable, protease-resistant structure similar to nucleoplasmin (see Supplementary Fig. 1). Chymotrypsin was used to selectively cleave the linker between GB1 and the NPL domain, allowing further purification.

**Fig. 3:**
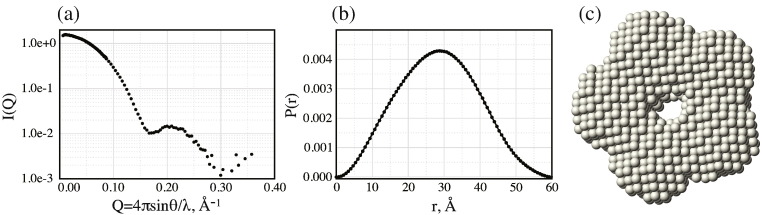
SANS of the NPL domain of FKBP39. (a) The scattering profile, (b) resulting distance distribution and (c) a beads model based on these data. The model was fitted with 5-fold symmetry. The data were collected on a deuterated sample of FKBP39. Each sphere of the model has a radius of 1.4 Å.

Size-exclusion chromatography gave an early indication that the NPL domain forms an oligomer, and the apparent molecular weight was consistent with the formation of a nucleoplasmin-like pentamer. In order to test this hypothesis, we studied deuterated NPL using small-angle neutron scattering (SANS). The SANS scattering profile matched a protein of about 50 kDa and produced an excellent fit when modelled with 5-fold symmetry. Modelling with a 6-fold symmetry did not fit the data so well. A bead model was constructed from the scattering profile (Fig. 3), and this clearly shows a doughnut-shaped structure that matches the dimensions of the nucleoplasmin pentamer.

**Fig. 4:**
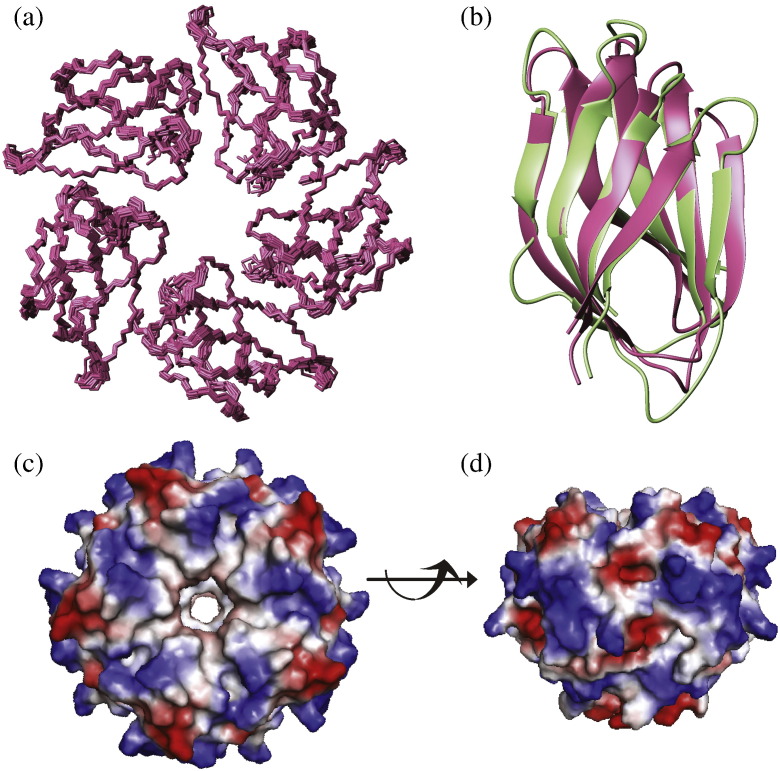
The structure of the FKBP39 NPL domain. (a) Superposition of 20 water-refined structures from the ensemble calculated from the final set of restraints. A 5-fold symmetry was enforced with non-crystallographic symmetry. (b) Cartoon representation of a monomer from the FKBP39 NPL structure (pink) superimposed with that from nucleoplasmin (1K5J, light green). The position of all β-strands is almost identical. (c) Electrostatic surface potential (− 60 to + 60 kT) viewed from the “top”, where the central pore is widest [same orientation as (a)]. (d) Side view of the same surface potential. The charges are quite evenly distributed over the surface.

Although the overall particle is large by NMR standards (54 kDa), it was possible to record good-quality 2D ^1^H-^15^N heteronuclear single quantum coherence (HSQC) spectra at ambient temperature. This encouraged us to determine the structure by NMR. With the aid of several isotope labelled samples, the structure of the NPL domain was determined. The ensemble of 20 water-refined structures is shown in Fig. 4a (structural statistics can be found in Supplementary Table 2). Solving the structure proved difficult because a mixed sample from proteins that had been isotope labelled in different ways was needed to assign inter-subunit nuclear Overhauser enhancements (NOEs), and it was not possible to reconstitute this due to the high stability of the NPL pentamer. This made it hard, and in some cases impossible, to assign inter-domain contacts (NOEs) close to the symmetry axis. For example, Ile56 contacts symmetry-related Ile56 residues in other subunits at the “bottom” of the central pore. Unambiguous assignment of a critical mass of inter-domain NOEs was eventually made with the aid of a deuterated sample that was selectively protonated on certain methyl groups and aromatic six-membered rings [16,17]. Once subunit contacts and the overall fold were determined, NOEs from other partially and fully protonated samples could be included to improve the structure.

Each monomer has a jelly-roll fold where the polypeptide chain folds back on itself to form an almost continuous antiparallel two-stranded ribbon. The β-ribbon is then twisted into 2 four-stranded sheets surrounding the hydrophobic core. The structures of nucleoplasmin (PDB ID 1K5J) and FKBP39 NPL are very similar, with a backbone root-mean-square difference for the structured part of 1.4 Å (Fig. 4b). All the β-strands occupy similar positions and only vary slightly in length. The same is true for the short loops—note that FKBP39 does not have any of the long loops seen in other members of this protein family—and all the residues in the loops are structured and restrained by experimental data. It is therefore clear that these two proteins share the same fold despite the low level of sequence similarity (12% identity). The subunits in the pentamer are tilted towards each other in a way that allows the hydrophobic core of one jelly-roll to interact with the outer hydrophobic surface of the next. The residues with the largest number of inter-domain NOEs are Phe4, Leu84 and Tyr47 (residue numbers of FKBP39 and the position of the β-strands are annotated in Fig. 2). None of these residues are strictly conserved. However, the hydrophobic character of Phe4 and Tyr47 is preserved. In addition, four hydrogen bonds (connecting His24 N^ε2^ on subunit E to Asp62 O^δ1^ on subunit A; Ile48 N on subunit A to Ser82 O^γ^ on subunit E; Thr51 N on subunit A to Gln58 O^ε1^ on subunit E; and Gln58 N^ε2^ on subunit E to Thr51 O on subunit A; and all the symmetry-related permutations) are formed at the interface between the subunits. With the exception of His24 and Asp62 that line the central pore (see below), none of the residues involved in hydrogen bonding are strictly conserved. As is the case with the hydrophobic contacts, there seems to be a variety of ways of establishing the subunit interface.

The first β-sheet of the FKBP39 NPL (strands β1, β8, β3 and β6, in structural order) features a β-bulge in its last strand at Val49 that is also present in NUP at the equivalent position, Ile78. Moreover, a second β-bulge at Ser26 (FKBP39) and Arg48 (NUP) is structurally conserved on the β3 strand—a very unusual feature since β3 is an inner strand of the sheet, hydrogen-bonding to two other strands. A third and final β-bulge is found in FKBP39's second sheet (strands β2, β7, β4 and β5) on β5 at Leu61 and Asp62. At the equivalent position in NUP, there is a larger bulge (Met91, Val92 and Gly93) due to the insertion of the glycine residue, which separates the two halves of the fifth strand. Thus, even unusual structural features are highly conserved between the two structures.

It is noteworthy that two of the bulges position Ser26 and Asp62 of FKBP39 at the heart of the central pore. These two residues are highly conserved, as are other residues on the surface of the pore (His24 and Tyr86). Thus, the central pore in FKBP39 (and most of other NPL domains) is lined with mostly conserved hydrophilic and charged residues. Given that few other surface residues are conserved across the alignment that are not required for the fold, the chemical environment of the central pore is the most defining feature of the structure, and we suspect that it may be functionally important. By contrast, the equivalent residues in NUP are emphatically different, resulting in a more hydrophobic and uncharged chemical environment. The most salient substitution is from Asp62 in FKBP39 (Asp in almost all the other sequences) to Val92 in NUP. It would therefore appear that NUP, though very similar in structure, is a protein with distinctly different properties to most NPL proteins.

### Comparison with other NPL domains

The properties of the outer surface (i.e., not the pore) of FKBP39 are not well conserved with respect to nucleoplasmin and other homologues, nor is the electrostatic surface potential (Fig. 4c and d). Our data suggest that the defining features of the NPL domains are the pentameric superstructure, the β-strand architecture supporting it, the chemical nature of the central pore and to a lesser degree the inter-subunit contacts.

**Fig. 5:**
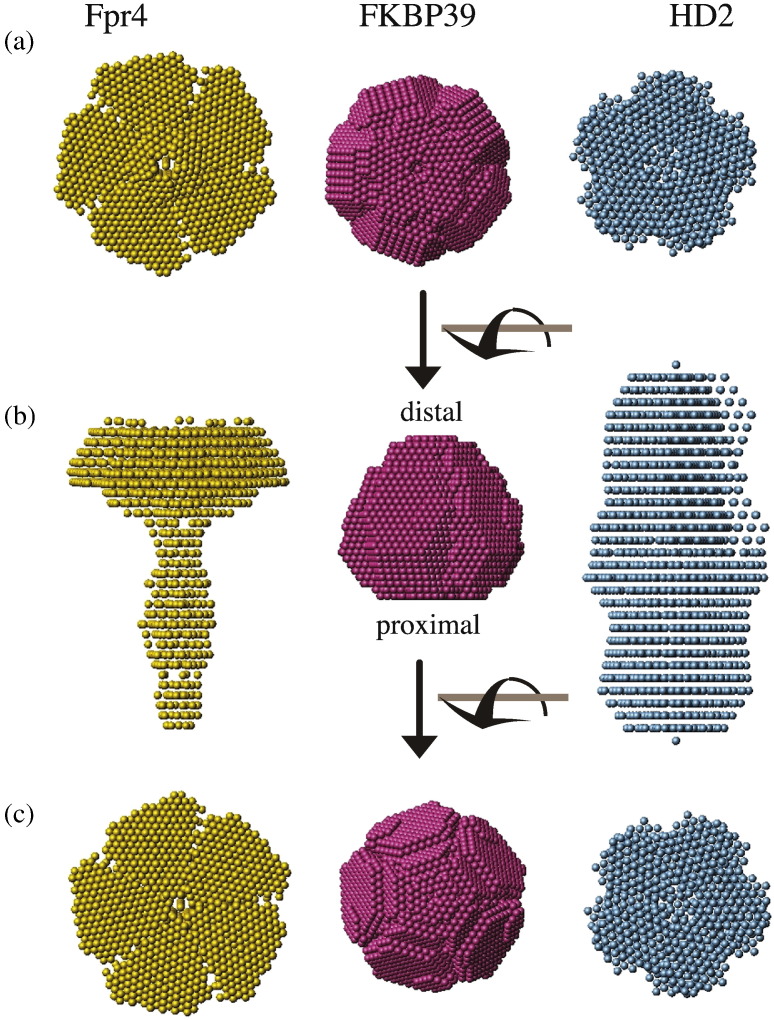
SAXS models of three NPL domains (a) top (proximal) view, (b) side view and (c) view from the bottom (distal) surface of the beads models of Fpr4 (yellow, left), FKBP39 (middle, pink) and HD2 (right, blue). The yeast Fpr4 NPL domain is the Δloop (residues 59–113 deleted) construct. The presence of the unstructured tail causes the particle to appear broader than the other two. The dimensions of the *Drosophila* FKBP39 SAXS model are very similar to the SANS model. This is a minimal NPL domain, much like the nucleoplasmin core. The SAXS model of *A*. *thaliana* HD2 is much larger and fits the dimensions of a decamer (a dimer of pentamers) very well. A sphere of a dummy atom has a radius of 1.4 Å. Fpr4 and FKBP39 were modelled with *P*5 symmetry; HD2 was modelled with *P*25 symmetry.

In order to test whether other NPL proteins also form similar oligomers, we expressed and purified two further NPL domains: yeast Fpr4 and *Arabidopsis* HD2. The Fpr4 sequence is only very weakly conserved (20% *versus* FKBP39; 12% *versus* NUP), while HD2 is much more similar to FKBP39 (36% identity). Interestingly, Fpr4 and other fungal NPL-FKBPs have a conserved Arg-x-Thr motif on β3 rather than the canonical His-x-Ser (His24, Ser26 in FKBP39), which has been hypothesised to be part of a catalytic site. Moreover, Fpr4 and many other fungal NPL-FKBPs contain an insertion of about 50 residues between the β4 and β5 strands, dividing the domain into two parts. In order to work with the domain, we removed this insertion by mutagenesis, leaving only the core domain. Small-angle X-ray scattering (SAXS) was employed to determine the size of the particle and its oligomeric state. As shown in Fig. 5, Fpr4 NPLΔloop formed a pentamer, confirming that one of the most distant cousins of nucleoplasmin has the same fold. This construct of Fpr4 contains a C-terminal “tail” that is clearly visible in the SAXS model. In addition, the unstructured tail results in a wider apparent “head” than those of the other two NPLs (Fig. 5). The disordered tail of nucleoplasmin has a similar effect on the SAXS model, increasing the apparent size of the particle substantially [18]. In summary, Fpr4's SAXS model is clearly consistent with the NPL fold.

The *Arabidopsis* NPL construct's SAXS model is also consistent with the NPL fold (Fig. 5). Fitting a dimer of nucleoplasmin (1K5J) to the scattering curve of HD2 resulted in an excellent fit, while doing the same with a monomer of NUP gave a poor result (Supplementary Fig. 3). Therefore, we modelled HD2 with *P*25 symmetry. The elongated model also includes flexible tails, which make the shape fuzzier than the excellent model of FKBP39. To our knowledge, this is the first time that a decamer has been observed in solution (SAXS studies of nucleoplasmin did not show any evidence of a homo-decamer [18]). We conclude that HD2 is also an NPL protein, however one that can dimerise under the conditions used in the SAXS experiment.

### FKBP39 binds to histones through its NPL domain

**Fig. 6:**
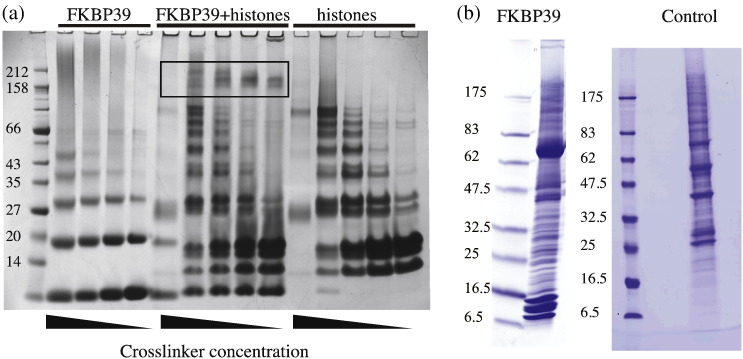
Histone binding and pull-down experiments. (a) FKBP39 NPL was cross-linked to a mixture of histones (octamers/dimers/tetramers) with BS^3^ (Pierce), a reagent that cross-links lysine residues. Both FKBP39 NPL (left) and histones (right) form intra-molecular cross-links, but high-molecular-weight cross-links between FKBP39 and histones can be seen in the range 150–200 kDa. (b) Full-length FKBP39 was tagged at the C-terminus with protein A and expressed in *Drosophila* D.Mel-2 cells. Cell lysates were bound to IgG beads and washed. The figure shows the eluate from the beads. The bait, FKBP39:protein A, runs at about 70 kDa. The most intense bands are in the low-molecular-weight range and correspond to the four core histones, which appear to be present in stoichiometric amounts. The control is the *Drosophila* kinetochore protein Nsl1, N-terminally tagged with protein A and purified in the same manner [23]. All gels are Coomassie stained.

Next, we addressed the question of whether FKBP39, like nucleoplasmin, may bind histones through its NPL domain. Figure 6a shows the results of an experiment where histone octamers and the FKBP39 NPL domain were incubated in the presence of a lysine-to-lysine cross-linker. Because both the histones and the NPL domain form internal cross-links, any cross-linked product between the two is most easily identified by comparison with the cross-linking observed with either the histones or NPL domain alone. Indeed, the presence of bands that are not observed in the lanes with either the histones or NPL domain alone must represent cross-linking between the two. The size of these cross-linked complexes is between 150 kDa and 200 kDa. These values are not easy to interpret because they do not necessarily represent the size of the native (non-covalent) complex. However, it can serve as a lower boundary. Assuming that each of these cross-links involves one pentamer, there would be between 100 and 150 kDa of histones in the complex—somewhere between 7 and 10 histones, depending on which histones are part of the complex. This would be consistent with the maximum load carried by NUP—10 histones or 5-histone H2A/H2B dimers [6]. In contrast, a more recent publication by the same group proposes a very distinct mode of NUP binding to H3/H4: in their model, one histone tetramer is capped by two NUP pentamers in a more prolate particle [19]. Both of these arrangements are consistent with our findings.

The cross-linking experiment does not identify which histones the NPL domain interacts with. We attempted to address this question using a peptide array and the Octet Red detection system. A tiled array of 96 histone peptides was assayed and the NPL domain was found to bind to a large number of them (data not shown). These included peptides from all four core histones. The array contained a large number of histone modifications, none of which seemed to specifically enhance binding. On balance, NPL exhibited an apparent preference for unmodified peptides.

In comparison with other proteins, the NPL domain does not form cross-links with histones very efficiently and the peptide binding data are in agreement with this observation: there does not seem to be a high-affinity interaction with any particular histone peptide. Rather, the results suggest moderate indiscriminate binding to all four core histones. As this behaviour may be moderated *in vivo*, we decided to also look at FKBP39's *in vivo* protein interactions.

### *In vivo* interactions of FKBP39

Using FKBP39 C-terminally fused to protein A as bait, we pulled down interacting proteins from *Drosophila* D.Mel-2 cells. After a single-step purification, protein samples were run on a gel and analysed by mass spectrometry. Examination of the gel immediately suggested that FKBP39 mainly binds histones (Fig. 6b, compare with control purification). All four core histones were confirmed to be present by mass spectrometry (see Supplementary Table 3). The intensity of the histone bands, and their purification in roughly stoichiometric amounts, suggests a direct interaction between FKBP39 and nucleosomes. A large number of other proteins were also identified by mass spectrometry. Most of these proteins were nuclear and indeed nucleolar by annotation, which is corroborated by our observation that endogenous FKBP39 localises strongly to the nucleolus (Supplementary Fig. 2), as do Fpr4 and Fpr3 [20]. Most prominent on the list are proteins from the small subunit processome (SSU), a large molecular factory involved in processing the ribosomal U3 RNA [21]. Although the sheer number of SSU proteins at the top of the list of identified proteins is strong evidence for an interaction with FKBP39, this might only be a consequence of FKBP39's nucleolar localisation. At present, we conclude that FKBP39 is a nucleolar protein that appears to interact with histones/chromatin and the SSU.

The C-terminal PPI of FKBP39, which has a pI of 10, did not show in our hands any interaction with histones *in vitro*. On the other hand, the PPI domain of Fpr4, which is similarly positively charged, does turn over H3 histone peptides and has a charge-compatible active site [12,14]. In order to establish which part of FKBP39 is responsible for the abovementioned interactions, two further protein A pull-down experiments were carried out: one with the amino-terminal half and the other one with the carboxy-terminal half of the protein (including the PPI domain). Only the N-terminal half of the protein that includes the NPL domain pulled down histones and other nucleolar proteins (Supplementary Tables 4 and 5). Because we found by mass spectrometry that the truncated N-terminal construct can form oligomers with endogenous full-length FKBP39 through the NPL, we can only conclude at present that it is not the C-terminal half by itself that is responsible for the interactions observed with the full-length protein. Thus, the NPL domain is necessary for interactions of FKBP39, although we cannot prove that it is sufficient due to its intrinsic ability to form oligomers with endogenous, full-length protein.

### Towards a function

As has been noted, most NPLs contain a histidine and an aspartate (His24 and Asp62 in FKBP39) that were thought to confer catalytic activity. A third residue, a serine (Ser26), is somewhat less conserved. In Fpr4, these residues are Arg-Thr-Asp instead of His-Ser-Asp; this triad is found in several other proteins, mostly from fungi. Since Arg cannot functionally substitute His in a catalytic triad, we assume *a priori* that the Arg-Thr-Asp NPLs must have a different function. Could the FKBP39 NPL domain be a protease, or more generally a hydrolase, possibly a lysine deacetylase? We addressed this question with different biochemical experiments. First, we asked whether the FKBP39 NPL domain is a histone deacetylase, as suggested by its homology to plant type 2 histone deacetylases [7–11]. We could not detect any HDAC activity using a commercial kit (Active Motif) that is based on a fluorescent acetyllysine analogue. The same result was obtained with the *Arabidopsis* HD2 NPL domain. Next, we tried generic assays for different enzyme activities, such as phosphatase, nuclease, protease, esterase, dehydrogenase and oxidase activity [22]. Again, no activity was found (data not shown).

**Fig. 7:**
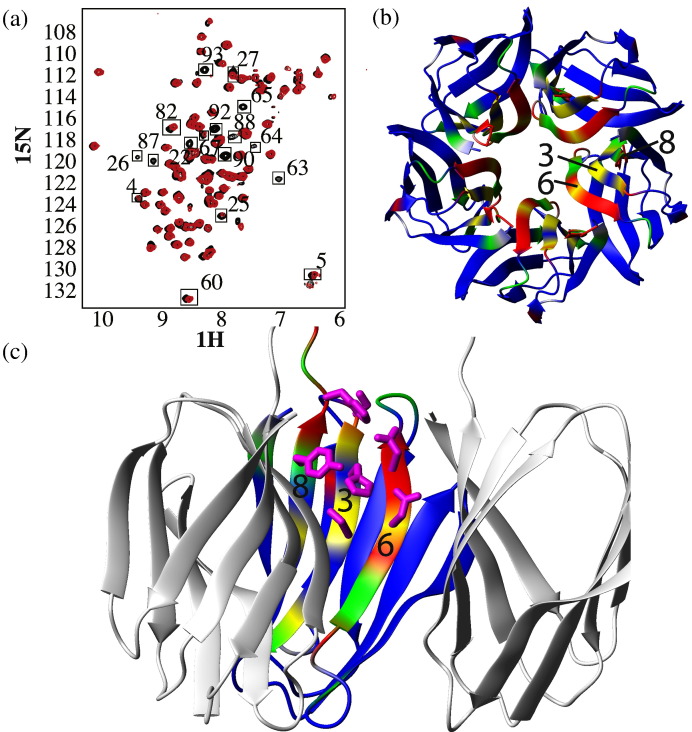
Divalent metal binding of FKBP39. FKBP39 binds to a panel of divalent metal ions with low affinity. In the 2D ^1^H,^15^N HSQC, (a) a subset of signals weaken or disappear on addition of metals. Their residue numbers are indicated. The reduction in peak volume was used to colour the ribbon representation of the FKBP39 pentamer from blue (no change) over green to red (strongly reduced) or grey where no data were available. (b) A top view of the pentamer and (c) a side view where the front two monomers have been removed. The side chains pointing into the pore of the most strongly affected areas are shown in purple. These are Tyr86 and His88 on β8; Ser22, His24 and Ser26 on β3; and Asp62 and Asn64 on β6.

Many enzymes require post-translation modifications, or association with a prosthetic group or activating protein. The only clue in any of these directions was an early observation that Mg^2 +^ further stabilised the already very stable protein in a thermal shift assay (Supplementary Fig. S1). Indeed, all the NMR experiments were conducted in the presence of 25 mM MgCl_2_ (and no other salt). To look at the role of different metal ions, we stripped the FKBP39 NPL domain with ethylenediaminetetraacetic acid and re-purified the protein to remove the chelator. Then, 2D ^1^H-^15^N HSQC experiments were recorded in the presence of each of several metal ions (usually chloride salts though the anion had no effect). The effect of divalent metal ions on the HSQC spectrum is strong (Fig. 7a) and very similar regardless of whether Cu(II), Co(II), Zn(II), Cd(II) or Mg(II) was used. Ca(II) had a weaker effect, which was comparable to 150 mM NaCl. In all of these spectra, the same cross-peaks diminished in intensity, while others stayed the same or became slightly more intense. The affected residues strongly correlate spatially and are located in the central pore and the region lining its exit on the more open side (Fig. 7b and c). Residues on the inward-facing β-strands include Ser22, His24 and Ser26 on β3; Asp62 and Asn64 on β6; and Tyr86 and His88 on β8. Any of these residues could in principal contribute to coordinating a metal ion. It ought to be noted that the most conserved residues, His24 and Asp62, are amongst the affected residues. Hence, their function, rather than being catalytic, may lie in metal binding. However, the promiscuous nature and relatively low affinity for a whole range of metals does not allow us to make more far-ranging inferences.

Thus, we conclude that the FKBP39 NPL domain binds divalent metal ions at the entrance or in the central pore relatively indiscriminately. The role of these ions—and the metal ligand bound *in vivo*—remains uncertain.

## Discussion

We have demonstrated that the N-terminal domain of *Drosophila* FKBP39 has a pentameric structure very similar to that of nucleoplasmin and that it can associate with histones both *in vivo* and *in vitro*. While the interaction with histones *in vivo* is strong (clear bands in stoichiometric amounts of all four core histones on a Coomassie-stained gel; Fig. 6a), the interaction *in vitro* appears to be relatively weak (Fig. 6b). This may suggest that the protein instead interacts with nucleosomes. In the *in vitro* binding experiment with histones, we worked with a minimal construct, a chymotrypsin-resistant fragment of the FKBP39 NPL domain. It should be noted that the interactions of histones with the nucleoplasmin core domain are also much weaker than with the native full-length and phosphorylated protein [18,19]. For example, the affinity of the *Xenopus* nucleoplasmin core domain for H2A/H2B is 15 × lower than the native protein [18]. Thus, the full-length FKBP protein may also show much higher affinity towards histones and nucleosomes *in vivo*.

FKBP39 is a nucleolar protein and we present evidence that it interacts *in vivo* with both nucleosomes and the small subunit processome (SSU). It has also been shown that FKBP39 co-purifies with several kinetochore proteins [23] and associates with microtubules [24]. However, no direct interaction with another protein has been reported. At the time of writing, publicly available interactomics/proteomics data (e.g., BioGRID 3.2 [25]) only show two yeast two-hybrid hits and none for affinity capture. One reason for this may be that the pentameric structure is incompatible with many protein engineering approaches. For instance, in our hands, only the GB1-tagged protein produced soluble expression in *Escherichia coli*, while other tags produced misfolded protein.

A closely related group of proteins, the so-called plant type 2 histone deacetylases or HD-tuins (Fig. 1), has been shown to have HDAC activity when purified from cell extracts [7–11]. We have shown that the *Arabidopsis* HD2 NPL domain adopts the same fold, as does that of yeast Fpr4, but we do not find any enzymatic activity; however, this could be due to the absence of a necessary co-factor, substrate or activating post-translational modification in our *in vitro* produced material. We have explored several avenues in a quest to identify an enzymatic activity. The most promising is the finding that the FKBP39 NPL domain binds divalent metal cations, some of which could support catalysis, for example, zinc. On the other hand, the conserved potentially catalytic residues in the HD2 and FKBP39 NPL domains are located in the central pore where they would be inaccessible to substrates, especially if a metal ion were bound. The 5-fold symmetry of the pore and the crowding of the putative active site residues (five histidines and five aspartates; five or ten serines), as well as the unfavourable geometry of any Ser-His-Asp catalytic triad, also make HDAC activity less likely. In summary, our data are more consistent with FKBP39, *Arabidopsis* HD2 and other related proteins not being histone deacetylases, or hydrolases.

Interestingly, our SAXS models show that HD2 forms a dimer of pentamers. Nucleoplasmin also crystallises as a decamer. Dimerisation is mediated by five hydrogen bonds that are semi-conserved [26] on the “closed” or “distal” surface. While the NUP decamer has not been observed in solution in the absence of binding partners, there is evidence that supports the formation of human Npm2 decamers in the presence of histones [26]. The two residues that promote dimerisation in the NUP crystal (Asp58 and Lys82) [5] are only semi-conserved within the NUP family, for example, Glu87 and Gln84 in human Npm2 [26]. The corresponding residues in HD2 are Gly38 and Ser58. While glycine is unsuitable to act as a hydrogen bond donor or acceptor, it is still conceivable that, given a suitable rearrangement of the loops and the bridging water molecules, a neighbouring residue, Lys36 or Glu39 for example, could take over that role. In some other NPLs, for example, the FKBP protein from the honeybee *Apis mellifera*, the dimer-promoting residues (Asp35 and Lys55) are fully conserved (Fig. 2, “A melli”). In *Drosophila* FKBP39, these residues are not conserved and the loops are shorter and hence conformationally more constrained. In summary, in some NPLs, the putative dimerisation-promoting residues are fully conserved; in others, they may be functionally conserved, while in yet others, they are absent.

**Fig. 8:**
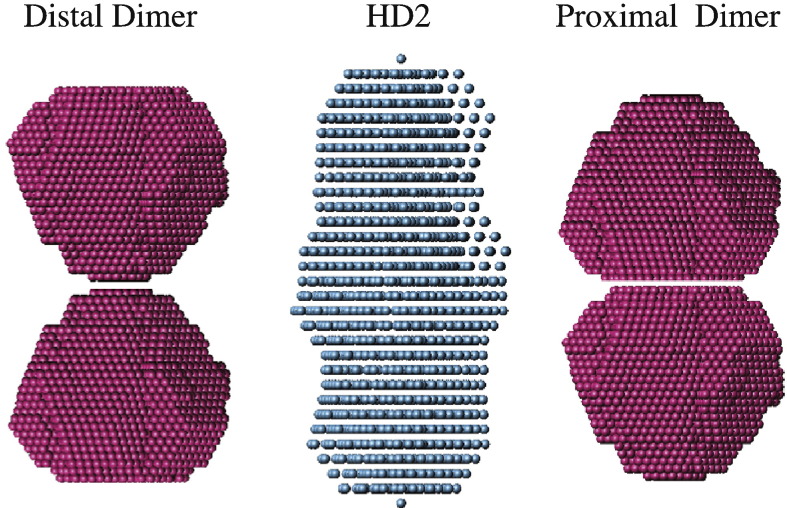
Two modes of HD2 NPL dimerisation. Dimerisation of the pentameric particle can occur via the distal or the proximal surface—the two modes are illustrated as dimers of the FKBP39 NPL SAXS model (pink). The HD2 model (middle) is rendered in blue.

Of course the dimer of pentamers could also be formed in a second way, via the “proximal” or “open” side of the pore (Fig. 8). Since the five N- and C-termini exit from the structured domain above the proximal surface, they would probably have to mediate the inter-dimer contacts. It is even conceivable that divalent metal ions contribute to dimer formation since they, too, bind to the proximal surface. Structurally, the biggest difference between the two modes of dimerisation is the positioning of potentially catalytic residues and the C-terminal tails. Distal dimerisation would place them at the ends of the long axis of the decamer. Proximal dimerisation, however, would place them at the equator. The potentially catalytic residues would be completely hidden in the proximal dimer, which could explain our failure to observe any enzymatic activity in HD2 (it does not, however, explain why FKBP39 shows no such activity).

Figure 8 illustrates the two possible dimerisation modes, distal and proximal. The SAXS model of HD2 is more consistent with the latter, as the width of the model increases at the equator, suggesting that additional mass (which can only be the tails) is located there. In contrast, no tail (cf. the Fpr4 model; Fig. 5) is visible at the poles. However, as our Fpr4 and the full-length nucleoplasmin SAXS models [18] show, the presence of unstructured tails (about 20 residues in HD2) can lead to disproportionate changes and distortions of a molecule's shape. With this in mind, our results should be considered with caution. Clearly more careful and detailed analyses are required to resolve this important question.

Yeast Fpr4, a protein with a split NPL domain and a variant “active site” (Arg-Asp instead of His-Asp), has been more thoroughly studied and shown to bind to the H2B nuclear localisation sequence [27], to function as a histone chaperone in nucleosome assembly [28], to be involved in regulating rDNA silencing [13] and to regulate lysine methylation and gene expression [12]. Interestingly, Nelson *et al.* report that Fpr4 directly binds histone H3 via the extreme H3 N-terminus, while the PPI domain acts on proline residues of H3 [12]. Many of these properties (binding to different histones, nucleolar localisation, the PPI domain) are shared by FKBP39, which may be a functional homologue. Our findings imply that Fpr4 also forms a pentamer *in vivo*, which opens up new possibilities as to how it might function. For instance, if Fpr4 were to bind to histone tails via its NPL domain, it would bring five PPI domains into close proximity with either histones or other chromatin complexes on the same or a neighbouring nucleosome. This increase in local concentration could boost PPI activity considerably or even lead to a PPI domain being bound to a target proline for as long as Fpr4/FKBP39 is attached to the respective nucleosome via its NPL.

Deletion of the FKBP39 gene in *Drosophila* and chromatin immuno-precipitation (ChIP-seq) has shown that FKBP39 is nucleolar, that it has a polycomb-like phenotype and that it may in some way positively regulate PRC2 [29]. The ChIP-seq data presented in the same study show that FKBP39 is present on chromatin throughout the genome without any particular pattern; however, these kinds of studies may not reflect differential chromatin localisation of FKBP39, which strictly depends on a particular physiological state of individual cells. All of these findings are in good agreement with our data that the NPL domain seems to confer the potential to interact with histones and chromatin. The fact that the FKBP39 knockout has a polycomb phenotype highlights the need for further research into the molecular function of NPL domains, which are conserved in a wide evolutionary context.

## Materials and Methods

### Constructs and cloning

All constructs of *Drosophila melanogaster* FKBP39 were generated from a cDNA source clone (ORF CG6226, UniProt P54397).

For *in vivo* pull-down experiments, cDNAs coding for the full-length protein and the N- and C-terminal halves (amino acids 1–357, 1–150 and 151–357, respectively) were cloned into a destination vector with a C-terminal protein A tag using gateway technology as described in Ref. [30]. For biochemical (*in vitro*) experiments, the N-terminal domain (residues 1–92) was cloned into a modified pET vector coding for an N-terminal His_6_-GB1-(tev) tag. The second amino acid of the open reading frame was changed from Ser to Ala to accommodate the NcoI cloning site.

The *Saccharomyces cerevisiae* Fpr4 (UniProt Q06205) β4-β5 loop deletion mutant was generated from construct amino acids 1–168 by overlap deletion PCR. Two fragments, coding for amino acids 1–58 and 114–168, were PCRed with an overlap of 30 nucleotides. The two PCR products were diluted 100-fold and combined in an overlap PCR step. The resulting fragment was cloned into a pET vector with a C-terminal His_6_ tag.

*Arabidopsis thaliana* type 2 histone deacetylase (HD2a, UniProt Q56WH4) was subcloned from a cDNA library into a pET N-His_6_-GB1-(tev) vector. The construct spanned amino acids 1–112.

### Recombinant protein expression in *E*. *coli* and isotopic labelling of the FKBP39 NPL domain

Recombinant protein was expressed in *E*. *coli* BL21 (DE3). ^15^N and ^15^N/^13^C labelling was carried out with standard protocols. All labelling schemes involving deuteration were carried out in the Life Sciences Group at the Institut Laue-Langevin in Grenoble. This group operates a platform that is now routinely used in support of neutron diffraction studies in crystallography, solution studies, reflectometry, fibre diffraction and NMR [31–41]. Cells were grown in 1-L fermenters with glycerol as sole carbon source. Precursors and inducer (1 mM IPTG) were added at appropriate times. Reverse ILV methyl labelling was carried out as previously described [16], using *d*_6_-glucose and [3,3-^2^H_2_,4-^13^C]-α-ketobutyrate and [3-^2^H,4,4-^13^C]-α-ketoisovalerate as precursors. Reverse ILV methyl labelling (as mentioned above) in combination with selective protonation of the six-membered rings of Phe, Tyr and Trp was achieved by using an auxotrophic strain and supplying shikimic acid before induction [17].

### Recombinant protein purification of FKBP39 NPL domain

The GB1 fusion expressed in the soluble fraction. Cell pellets were resuspended in lysis buffer [20 mM Tris–HCl (pH 8.0), 150 mM, 10 mM imidazole and 2 mM β-mercaptoethanol] supplemented with 1 mM PMSF. Cell lysis was achieved by adding lysozyme and sonication. DNase I and 5 mM MgCl_2_ were also added. After centrifugation, the soluble fraction was applied to a hand-held IMAC column. After extensive washes, the His-tagged protein was eluted with lysis buffer plus 200 mM imidazole. All fractions containing the protein were pooled and concentrated to approximately 20 mg/mL. The GB1 tag was then cleaved off by adding 1:100 (w/w) chymotrypsin and incubating at 37 °C or room temperature for 1 h. Overdigestion did not result in internal cleavage of the FKBP39 NPL domain. After digestion, samples were centrifuged to remove precipitate and applied to a S200 or S75 120-mL column, equilibrated with lysis buffer minus imidazole. FKBP39 NPL domain eluted in a single symmetric peak. Fractions were pooled and concentrated to up to 20 mg/mL (~ 2 mM in monomer or 0.4 mM pentamer). The identity of the protein was confirmed by mass spectrometry. After chymotrypsin cleavage, the protein contained 3–4 additional amino acids ([F]QGA) at the N-terminus and the dipeptide GS (BamHI site) at the C-terminus. For NMR experiments, the protein was exchanged into 20 mM sodium phosphate buffer (pH 6.0) and 25 mM MgCl_2_ and was concentrated to volumes between 150 and 500 μL and 0.5–1 mM (monomer) concentration. Samples in D_2_O were prepared by lyophilising and resuspending in 99.9% D_2_O twice. SANS and SAXS conditions were identical with NMR conditions. To test the effect of different metal ions on NMR spectra, we first treated a ^15^N-labelled sample with 10 mM ethylenediaminetetraacetic acid and then buffer exchanged it via size-exclusion chromatography into either 50 mM Tris (pH 8.0) or 50 mM 4-morpholineethanesulfonic acid (pH 6.0). After recording a reference spectrum, we added a concentrated solution of metal salts to a final concentration of 1 mM.

### Protein A pull-down, purification and mass spectrometry of FKBP39 full-length and truncations

Protein A affinity purifications from cell lysates and mass spectrometry were carried out essentially as previously described [30]. Briefly, protein-A-tagged proteins were expressed in *Drosophila* D.Mel-2 cells. Cleared cells lysates were bound to DynaBeads (Invitrogen) conjugated to rabbit IgG (MP Biomedicals) and eluted with 0.5 M NH_4_OH after washing. Samples were analysed by SDS-PAGE and liquid chromatography tandem mass spectrometry. Protein samples were digested with trypsin (Promega V5111) following standard procedures. Peptide mixtures were analysed by liquid chromatography/mass spectrometry using a Nano-Acquity (Waters) LC system and Orbitrap Velos mass spectrometer (Thermo Electron Corp., San Jose, CA). Acquired spectra were processed by Mascot Distiller followed by a Mascot search (Matrix Science) against the FlyBase database. Proteins with a Mascot score above 50 with at least two identified peptides were considered to be positively identified.

### X-linking

FKBP39 to histone octamer cross-linking was carried out with BS^3^ cross-linker (Pierce) in 20 mM Hepes (pH 7.5), 150 mM NaCl and 1 mM DTT. Freshly prepared 3% (w/v) BS^3^ stock solution was diluted 1/5, 1/10, 1/20, 1/50 and 1/100 with distilled water, and 1 μL of the resulting solution was mixed with 10 μL of ~ 10 μM FKBP39 alone, histone octamers (Abcam) alone or FKBP39 with octamers, prior to incubation at room temperature for 30 min. Cross-linking was quenched by adding 1 μL of 1 M Tris (pH 8.0) to each reaction. Samples were analysed by SDS-PAGE.

### SANS and SAXS

SANS experiments were carried out as described in Ref. [41]. Experiments were conducted on beamline D22 of the Institut Laue-Langevin, Grenoble. Samples were measured at a temperature of 20 °C and at detector distances of 4 and 14 m, covering an overall *Q* range of 0.0034 < *Q* < 0.143 Å^− 1^ (where *Q* = 4πsinθ/λ). The samples were prepared in concentration ranges from 2.0 to 10.0 mg/mL in 20 mM 4-morpholineethanesulfonic acid (pH 6.0) and 25 mM NaCl.

SAXS data of Fpr4Δloop were collected at the X33 beamline at the European Molecular Biology Laboratory/Deutsches Elektronen-Synchrotron (Hamburg) at a temperature of 11 °C using a camera length of 2.7 m covering a range of momentum transfer 0.08 ≤ *Q* ≤ 6.0 nm^− 1^ (*Q* = 4πsinθ/λ, where 2θ is the scattering angle and λ = 0.15 nm is the X-ray wavelength). The samples were prepared in concentration ranges from 1.0 to 4.0 mg/mL in 50 mM Hepes (pH 7.5) and 100 mM NaCl.

SAXS experiments of HD2 and FKBP39 NPLs were conducted at the European Synchrotron Radiation Facility BioSAXS beamline ID14eh3 [42] in Grenoble (France). Samples were loaded into the measurement cell and exposed to X-rays, and scattering data were collected using the robotic sample handling available at the beamline [43], at a temperature of 11 °C using a camera length of 2.8 m covering a range of momentum transfer 0.08 ≤ *Q* ≤ 5 nm^− 1^ (*Q* = 4πsinθ/λ, where 2θ is the scattering angle and λ = 0.931 nm is the X-ray wavelength). Samples were prepared in concentration ranges from 1.0 to 5.0 mg/mL (HD2 NPL) and from 1.0 to 20.0 mg/mL (FKBP39 NPL) in 50 mM Hepes (pH 7.5) and 100 mM NaCl.

SANS and SAXS data were processed in a similar fashion. The *P*(*r*) distance distribution, maximum dimension *D*_max_ and radius of gyration *R*_g_ were obtained using the programmes PRIMUS [44] and GNOM [45], and the *ab initio* models were generated with GASBOR [46] or DAMMIF [47] and then averaged, aligned and compared using DAMAVER [48]. The fits to the experimental data of the models and the theoretical scattering of the structures were calculated with CRYSOL [49] and figures were prepared with SAXSVIEW†.

### NMR spectroscopy of FKBP39 NPL domain and structure determination

All spectra were processed with NMRPipe [50] and spectral analysis was carried out with NMRView [51] and CcpAnalysis [52].

Backbone experiments of ^2^H/^13^C/^15^N-labelled protein were carried out using transverse relaxation optimised spectroscopy (TROSY) versions of the HNCA, HN(CA)CB, HN(CO)CA and HN(COCA)CB on a Bruker 800-MHz spectrometer equipped with a cryoprobe. Sidechain resonances were partially assigned with 3D HCCH total correlated spectroscopy (TOCSY) and correlated spectroscopy (COSY) experiments on a double (^15^N/^13^C)-labelled sample. Both backbone and side-chain assignment relied heavily on 3D NOESY spectra. NOE peaklists together with dihedral restraints derived from backbone chemical shifts using Dangle [53] served as input to ARIA2 [54]. First, only monomer structures were calculated. Once inter-domain NOEs could be assigned, the full pentamer was calculated in ARIA2 applying *C*5 symmetry. In the final iteration, 100 structures were calculated and the 20 best were refined in explicit water. The coordinates of the ensemble of water-refined structures were deposited at the PDB. Statistics on the structural ensemble can be found in Supplementary Table 2. Figures were generated with PyMOL (Schrödinger) and MOLMOL [55].

## Appendix A Supplementary data

Supplementary Table 1 Proteins used in the phylogenetic tree.

Supplementary Table 2 Structural statistics of the NMR ensemble.

Supplementary Table 3 Proteomics table of identified proteins in the FKBP39 full-length pull-down.

Supplementary Table 4 Proteomics table of identified proteins in the FKBP39 1-150 (N-terminal half) pull-down.

Supplementary Table 5 Proteomics table of identified proteins in the FKBP39 151-357 (C-terminal half) pull-down.

Supplementary Figure1: thermal shift assay of FKBP39 NPL. Supplementary Figure2: subcellular localisation of FKBP39. Supplementary Figure3: SAXS scattering curves of FKBP39 and HD2 NPLs. Supplementary methods (thermal shift, microscopy).

## Footnotes

† http://saxsview.sourceforge.net.
