# Supplementary material for: The Pentameric Nucleoplasmin Fold Is Present in Drosophila FKBP39 and a Large Number of Chromatin-Related Proteins
Source: J Mol Biol. 2015 May 22;427(10):1949–63. doi: 10.1016/j.jmb.2015.03.010 (PMC4414354; doi:10.1016/j.jmb.2015.03.010)
Supplement: Supplementary Table 1 — Proteins used in the phylogenetic tree. [file mmc1.pdf]

Supplementary Table 1 - phylogeny

Supplementary methods for generating the phylogenetic tree

HMMER[1] online[a] was run with standard parameters (E-value cutoff of 0.03) and the first 93 residues of *Drosophila melanogaster* FKBP39 protein as input against the "Uniprot reference proteome" database. The search was repeated three times with all hits above threshold of the previous rounds contributing to the profile of the following run. Protein sequences from a selection of representative species were downloaded, aligned again with Mafft[2] and further edited by hand based on the structural alignment between FKBP39 and *Xenopus* nucleoplasmin (1KJ5). Columns with more than 90% gaps were removed with a Python script. Pairwise distances were calculated and a tree constructed using the Philip[3] neighbour-joining algorithm. The resulting unrooted tree was visualised and plotted with DrawTree (part of the Phylip package).

[a]url:http://hmmer.janelia.org/search/hmmsearch

	Uniprot.ID	Species	Common.name	Function	Kingdom	ID
1	A0NDN1_ANOGA	Anopheles gambiae	mosquito	nucleoplasmin	Animals	ANOGA1
2	Q7QJE1_ANOGA	Anopheles gambiae	mosquito	NPL-FKBP	Animals	ANOGA2
3	H9KHI9_APIME	Apis mellifera	honey bee	NPL-FKBP	Animals	APIME1
4	H9KH00_APIME	Apis mellifera	honey bee	nucleoplasmin	Animals	APIME2
5	E1C540_CHICK	Gallus gallus	chicken	nucleoplasmin	Animals	CHICK1
6	NLP_DROME	Drosophila melanogaster	fruit fly	nucleoplasmin	Animals	DROME1
7	Q9VAC4_DROME	Drosophila melanogaster	fruit fly	nucleoplasmin	Animals	DROME2
8	FKB39_DROME	Drosophila melanogaster	fruit fly	NPL-FKBP	Animals	DROME3
9	F7CKP2_HORSE	Equus caballus	horse	nucleoplasmin	Animals	HORSE1
10	K9K3J3_HORSE	Equus caballus	horse	nucleoplasmin	Animals	HORSE2
11	NPM3_HUMAN	Homo sapiens	human	nucleoplasmin	Animals	HUMAN1
12	NPM_HUMAN	Homo sapiens	human	nucleoplasmin	Animals	HUMAN2
13	F6VS63_ORNAN	Ornithorhynchus anatinus	platypus	nucleoplasmin	Animals	ORNAN1
14	F6YUB8_ORNAN	Ornithorhynchus anatinus	platypus	nucleoplasmin	Animals	ORNAN2
15	E0VJG3_PEDHC	Pediculus humanus	louse	NPL-FKBP	Animals	PEDHC1
16	E0V9N0_PEDHC	Pediculus humanus	louse	nucleoplasmin	Animals	PEDHC2
17	NPM_RAT	Rattus norvegicus	rat	nucleoplasmin	Animals	RAT1
18	F7FKF2_RAT	Rattus norvegicus	rat	nucleoplasmin	Animals	RAT2
19	Q7M6Z1_RAT	Rattus norvegicus	rat	nucleoplasmin	Animals	RAT3
20	Q4SCS8_TETNG	Tetraodon nigroviridis	pufferfish	nucleoplasmin	Animals	TETNG1
21	Q28FE3_XENTR	Xenopus tropicalis	frog	nucleoplasmin	Animals	XENTR1
22	F7DW01_XENTR	Xenopus tropicalis	frog	nucleoplasmin	Animals	XENTR2
23	FKBP3_CANAL	Candida albicans		NPL-FKBP	Fungi	CANAL1
24	FKBP4_CRYNJ	Cryptococcus neoformans		NPL-FKBP	Fungi	CRYNJ1
25	FKBP4_NEUCR	Neurospora crassa		NPL-FKBP	Fungi	NEUCR1
26	FKBPH_SCHPO	Schizosaccharomyces pombe	fission yeast	NPL-FKBP	Fungi	SCHPO1
27	FKBP4_SCHPO	Schizosaccharomyces pombe	fission yeast	NPL-FKBP	Fungi	SCHPO2
28	FKBP4_YEAST	Saccharomyces cerevisiae	baker's yeast	NPL-FKBP	Fungi	YEAST1
29	FKBP3_YEAST	Saccharomyces cerevisiae	baker's yeast	NPL-FKBP	Fungi	YEAST2
30	HD-tuinT4_ARATH	Arabidopsis thaliana	thale cress		Plants	ARATH1
31	HD-tuinT3_ARATH	Arabidopsis thaliana	thale cress	HD-tuin	Plants	ARATH2
32	HD-tuinT1_ARATH	Arabidopsis thaliana	thale cress	HD-tuin	Plants	ARATH3
33	HD-tuinT2_ARATH	Arabidopsis thaliana	thale cress		Plants	ARATH4
34	FKB53_ARATH	Arabidopsis thaliana	thale cress	NPL-FKBP	Plants	ARATH5
35	FKB43_ARATH	Arabidopsis thaliana	thale cress	NPL-FKBP	Plants	ARATH6
36	A8IYI1_CHLRE	Chlamydomonas reinhardtii	algae		Protists	CHLRE
37	A9P996_POPTR	Populus trichocarpa	poplar	HD-tuin	Plants	POPTR1
38	B9HG89_POPTR	Populus trichocarpa	poplar		Plants	POPTR2
39	A9P950_POPTR	Populus trichocarpa	poplar	HD-tuin	Plants	POPTR3
40	B9MTN7_POPTR	Populus trichocarpa	poplar	HD-tuin	Plants	POPTR4
41	K4D3Z0_SOLLC	Solanum lycopersicum	tomato	HD-tuin	Plants	SOLLC1
42	K4D9U1_SOLLC	Solanum lycopersicum	tomato		Plants	SOLLC2
43	K4AZ74_SOLLC	Solanum lycopersicum	tomato	NPL-FKBP	Plants	SOLLC3
44	K4CQT3_SOLLC	Solanum lycopersicum	tomato	HD-tuin	Plants	SOLLC4
45	K4BEE0_SOLLC	Solanum lycopersicum	tomato	NPL-FKBP	Plants	SOLLC5
46	D7T228_VITVI	Vitis vinifera	wine grape	NPL-FKBP	Plants	VITVI1
47	D7TQZ5_VITVI	Vitis vinifera	wine grape	NPL-FKBP	Plants	VITVI2
48	D7SN59_VITVI	Vitis vinifera	wine grape	HD-tuin	Plants	VITVI3
49	F6HKH5_VITVI	Vitis vinifera	wine grape	HD-tuin	Plants	VITVI4

Table 1: Proteins used to generate the phylogenetic tree in Fig. 1. Plant proteins that are not functionally annotated are in all cases HD-tuins where at least one of the coordinating residues in the zinc finger is mutated.

	Species	Common name	KIngdom	Proteins
1	Anopheles gambiae	mosquito	Animals	2
2	Apis mellifera	honey bee	Animals	2
3	Arabidopsis thaliana	thale cress	Plants	6
4	Candida albicans		Fungi	1
5	Chlamydomonas reinhardtii	algae	Protists	1
6	Cryptococcus neoformans		Fungi	1
7	Drosophila melanogaster	fruit fly	Animals	3
8	Equus caballus	horse	Animals	2
9	Gallus gallus	chicken	Animals	1
10	Homo sapiens	human	Animals	2
11	Neurospora crassa		Fungi	1
12	Ornithorhynchus anatinus	platypus	Animals	2
13	Pediculus humanus	louse	Animals	2
14	Populus trichocarpa	poplar	Plants	4
15	Rattus norvegicus	rat	Animals	3
16	Saccharomyces cerevisiae	baker's yeast	Fungi	2
17	Schizosaccharomyces pombe	fission yeast	Fungi	2
18	Solanum lycopersicum	tomato	Plants	5
19	Tetraodon nigroviridis	pufferfish	Animals	1
20	Vitis vinifera	wine grape	Plants	4
21	Xenopus tropicalis	frog	Animals	2

Table 2: Summary of species in Table 1. CHLRE is a unicellular alga which are sometimes classified as plants and sometimes as protists.

References

[1] Eddy, S. R. (2011). Accelerated Profile HMM Searches. PLoS Comput. Biol. 7, e1002195.

[2] Katoh, K., & Standley, D. M. (2013). Mafft multiple sequence alignment software version 7: Improvements in performance and usability. Mol. Biol. and Evol. 30, 772–780.

[3] Farris, J. S. (1989). Notices. Cladistics 5, 163–166.
