# Supplementary material for: The Pentameric Nucleoplasmin Fold Is Present in Drosophila FKBP39 and a Large Number of Chromatin-Related Proteins
Source: J Mol Biol. 2015 May 22;427(10):1949–63. doi: 10.1016/j.jmb.2015.03.010 (PMC4414354; doi:10.1016/j.jmb.2015.03.010)
Supplement: Supplementary Table 2 — Structural statistics of the NMR ensemble. [file mmc2.pdf]

Structural Statistics for FKBP39

Experimental Restraints per Monomer	
Unambiguous NOEs	822
Ambiguous NOEs	173
Dihedral angle restraints (phi & psi)	159
Hydrogen bonds	90
Long-range NOEs	795
Intermolecular NOEs	59

Structural Statistics Residues 3-91		
	<SA>[1]	<SA>$_c$[2]
**Coordinate Precision (Å)**		
Backbone	0.63	0.62
Heavy atoms	1.05	1.02
**Ramachandran analysis (%)**		
Core	86.0	87.7
Allowed	13.8	12.1
Generously allowed	0.2	0.2
Disallowed	0.1	0.0
**RMS deviation from restraints**		
NOE distances (Å)	0.60	0.59
Dihedral angles (°)	0.40	0.37
**RMS deviation from idealised geometry**		
bonds (Å)	0.0045	0.0046
Angles (°)	0.57	0.55
impropers (°)	1.86	1.79

[1]<SA> is the average for the ensemble of 20 water-refined structures
[2]<SA>$_c$ is the structure closest to the mean
