# Supplementary material for: The Pentameric Nucleoplasmin Fold Is Present in Drosophila FKBP39 and a Large Number of Chromatin-Related Proteins
Source: J Mol Biol. 2015 May 22;427(10):1949–63. doi: 10.1016/j.jmb.2015.03.010 (PMC4414354; doi:10.1016/j.jmb.2015.03.010)
Supplement: Supplementary Table 4 — Proteomics table of identified proteins in the FKBP39 1-150 (N-terminal half) pull-down. [file mmc4.pdf]

FKBP39_1-150_CprA

CG	Size	Score	# of peptides	Full Name	Function
CG7439	137677	1558	59	Argonaute 2	
CG6226	39376	1545	81	FKBP39 (bait)	
CG4264	71372	1519	64	Heat shock protein cognate 4	
CG6501	76674	658	25	Ngp	
CG5436	70043	509	16	Heat shock protein 68	
CG18743	70516	509	16	Heat-shock-protein-70Ab	
CG4147	72330	428	13	Heat shock protein cognate 3	
CG7808	23859	389	9	Ribosomal protein S8	
CG4389	84478	386	13		
CG5502	45112	315	14	Ribosomal protein L4	
CG16973	121355	313	11	misshapen	Signalling
CG6875	231517	296	10	abnormal spindle	
CG10360	65776	282	12	refractory to sigma P	
CG3751	15095	276	7	Ribosomal protein S24	
CG9277	50571	269	12	beta-Tubulin at 56D	
CG10805	238952	264	9	UTP10 homolog	small-subunit processome
CG12819	158956	260	7	slender lobes	nucleolus
CG17498	23618	255	7	mad2	spindle checkpoint
CG4463	20730	254	10	Heat shock protein 23	
CG14648	59934	253	8		
CG12261	45873	249	11	mitochondrial ribosomal protein S22	
CG9282	17623	239	5	Ribosomal protein L24	
CG4863	45806	235	6	Ribosomal protein L3	
CG8064	104226	208	6	UTP12, DIP2 homolog	small-subunit processome
CG12301	88731	205	5	UTP14 homolog	small-subunit processome
CG2512	50486	204	7	alpha-Tubulin at 84D	
CG8280	50561	190	12	Elongation factor 1alpha48D	
CG6253	19219	180	4	Ribosomal protein L14	
CG5728	160619	170	5	RRP5 homolog	small-subunit processome
CG7728	133752	169	5	BMS1 homolog	ribosome assembly
CG4466	23659	164	9	Heat shock protein 27	
CG9799	104155	162	7	UTP21 homolog	small-subunit processome
CG12775	18521	161	5	Ribosomal protein L21	
CG8857	18265	161	5	Ribosomal protein S11	
CG6815	68489	160	5	belphegor	
CG4183	23208	158	6	Heat shock protein 26	
CG2168	30549	154	3	Ribosomal protein S3A	
CG3195	17891	154	3	Ribosomal protein L12	
CG12396	79289	150	5	NNP-1 homolog, RRP1-like protein	rRNA processing
CG1489	45999	142	2	Pros45	
CG1671	89564	142	4	UTP13 homolog	small-subunit processome
CG1994	113659	139	3	p110, GCN5 related	PRC1 complex
CG4554	313818	137	6	UTP20 homolog	small subunit processome
CG7490	34295	136	4	Ribosomal protein LP0	
CG9300	76766	136	4	NOL11 homolog	nucleolus
CG13849	55031	135	3	Nop56	small subunit processome
CG1263	27989	135	3	Ribosomal protein L8	
CG4581	51009	134	6	Thiolase	
CG3401	51387	131	6	beta-Tubulin at 60D	

CG	Size	Score	# of peptides	Full Name	Function
CG1883	22156	130	5	Ribosomal protein S7	
CG8863	45886	129	3		
CG10652	12398	129	4	Ribosomal protein L30	
CG3379	11374	129	5	Histone H4	nucleosome
CG4202	49538	124	3	UTP3 homolog Sas10	small subunit processome
CG10944	28882	124	6	Ribosomal protein S6	
CG1542	35056	123	2	Ebp2	rRNA processing
CG7246	70572	122	4	UTP6 homolog	small-subunit processome
CG11276	29230	120	5	Ribosomal protein S4	
CG2960	15005	119	6	Ribosomal protein L40	
CG32253	41408	117	2	BRX1 homolog	ribosomal large subunit biogenesis
CG12050	111364	116	2	UTP17, WDR75	small-subunit processome
CG6937	39594	116	3		RNA binding
CG8900	17658	116	3	Ribosomal protein S18	
CG9888	34673	113	3	Fibrillarin	small subunit processome
CG17870	28379	110	2	14-3-3zeta	
CG8922	25760	109	2	Ribosomal protein S5a	
CG16944	33116	109	3	stress-sensitive B	
CG13097	76056	108	5	Mpp10 superfamily	small subunit processome
CG7434	30649	107	4	Ribosomal protein L22	
CG3949	14045	102	2	hoi-polloi	small subunit processome
CG3661	15041	102	5	Ribosomal protein L23	
CG9688	16406	101	4	mitochondrial ribosomal protein S18C	
CG3983	66457	101	5		
CG30349	68663	99	4	UTP5 homolog	small-subunit processome
CG32031	61511	98	5	Arginine kinase	arginine kinase activity
CG33505	53823	97	2	U3-55k, RRP9 homolog	rRNA processing
CG3395	22610	97	5	Ribosomal protein S9	
CG4258	39989	96	2	dribble, KRR1 small subunit processome component homolog	small subunit processome
CG31022	63360	95	2	prolyl-4-hydroxylase-alpha EFB	
CG3071	60559	95	2	UTP15 superfamily	small subunit processome
CG11999	23847	95	2		membrane
CG8849	29198	93	2	mitochondrial ribosomal protein L24	
CG7977	29445	91	3	Ribosomal protein L23A	
CG7057	50109	90	2	AP-50	
CG40049	46298	89	2	mitochondrial ribosomal protein S5	
CG31256	74341	88	2	Brf	
CG6779	27682	87	2	Ribosomal protein S3	
CG31618	13355	87	3	Histone H2A	Nucleosome
CG1821	14581	84	2	Ribosomal protein L31	
CG2998	7529	83	2	Ribosomal protein S28b	
CG4027	42194	81	3	Actin 5C	
CG5650	35260	80	2	Protein phosphatase 1 at 87B	
CG4260	106352	80	2	alpha-Adaptin	
CG12792	51166	78	2	lethal (2) 09851	

FKBP39_1-150_CprA

CG	Size	Score	# of peptides	Full Name	Function
CG1475	23803	74	2	Ribosomal protein L13A	
CG3428	36006	74	3		
CG1524	16312	73	3	Ribosomal protein S14a	
CG4364	74045	72	2	Pescadillo homolog	rRNA processing
CG12325	107064	69	2	UTP1 homolog	small-subunit processome
CG6846	17270	69	3	Ribosomal protein L26	
CG13389	17168	67	2	Ribosomal protein S13	
CG12785	136870	67	2	UTP22 homolog, Mat988Ba, NOL6	small-subunit processome
CG7610	33078	64	2	ATP synthase-gamma chain	
CG8615	21822	64	2	Ribosomal protein L18	
CG10596	70619	61	2	Msr-110	
CG5148	110554	59	2		
CG1142	32732	59	2	Fcf2 superfamily	rRNA processing
CG18174	34550	58	2	Rpn11	
CG1651	172000	58	2	Ankyrin	
CG8415	16064	58	2	Ribosomal protein S23	
CG10206	57477	58	2	UTP22, NOL6, NOP5	small-subunit processome
CG18001	8252	55	2	Ribosomal protein L38	
CG3251	58063	55	2		
CG3132	72137	54	2	Ect3	
CG33555	165412	51	2	bitesize	

small sububit processome

also present in full-length pull-down
