# Supplementary material for: The Pentameric Nucleoplasmin Fold Is Present in Drosophila FKBP39 and a Large Number of Chromatin-Related Proteins
Source: J Mol Biol. 2015 May 22;427(10):1949–63. doi: 10.1016/j.jmb.2015.03.010 (PMC4414354; doi:10.1016/j.jmb.2015.03.010)
Supplement: Supplementary Table 5 — Proteomics table of identified proteins in the FKBP39 151-357 (C-terminal half) pull-down. [file mmc5.pdf]

FKBP39_151-357_CprA

CG #	Size	Score	#of peptides	Full name
CG8947	39376	1106	59	FK506-binding protein 1
CG11999	71372	801	40	Heat shock protein cognate 4
CG7808	50571	754	41	beta-Tubulin at 56D
CG10652	72330	386	13	Heat shock protein cognate 3
CG15792	50486	287	10	alpha-Tubulin at 84D
CG2411	51387	242	12	beta-Tubulin at 60D
CG10377	62622	227	4	
CG2168	60885	212	5	Heat shock protein 60
CG2135	42072	150	5	Actin 88F
CG3195	23618	136	5	mad2
CG2960	17891	115	3	Ribosomal protein L12
CG32031	107009	111	5	
CG31915	62661	101	2	26-29kD-proteinase
CG10824	51030	87	5	Elongation factor 1alpha100E
CG12101	23847	86	2	
CG5178	15005	82	3	Ribosomal protein L40
CG17498	23859	82	2	Ribosomal protein S8
CG31198	12398	81	2	Ribosomal protein L30
CG1873	61511	64	3	Arginine kinase
CG4957	237395	64	2	zipper
CG2512	71446	63	3	
CG3401	144051	62	2	patched
CG4147	44970	61	2	Heterogeneous nuclear ribonucleoprotein at 27C
CG4264	30549	59	2	Ribosomal protein S3A
CG9277	79480	52	2	
CG6226	27749	50	5	

also present in full-length pull-down
