# Supplementary material for: The Pentameric Nucleoplasmin Fold Is Present in Drosophila FKBP39 and a Large Number of Chromatin-Related Proteins
Source: J Mol Biol. 2015 May 22;427(10):1949–63. doi: 10.1016/j.jmb.2015.03.010 (PMC4414354; doi:10.1016/j.jmb.2015.03.010)
Supplement: Supplementary file 6 — Supplementary Figure1: thermal shift assay of FKBP39 NPL. Supplementary Figure2: subcellular localisation of FKBP39. Supplementary Figure3: SAXS scattering curves of FKBP39 and HD2 NPLs. Supplementary methods (thermal shift, microscopy). [file mmc6.pdf]

Supplementary Figures and Methods

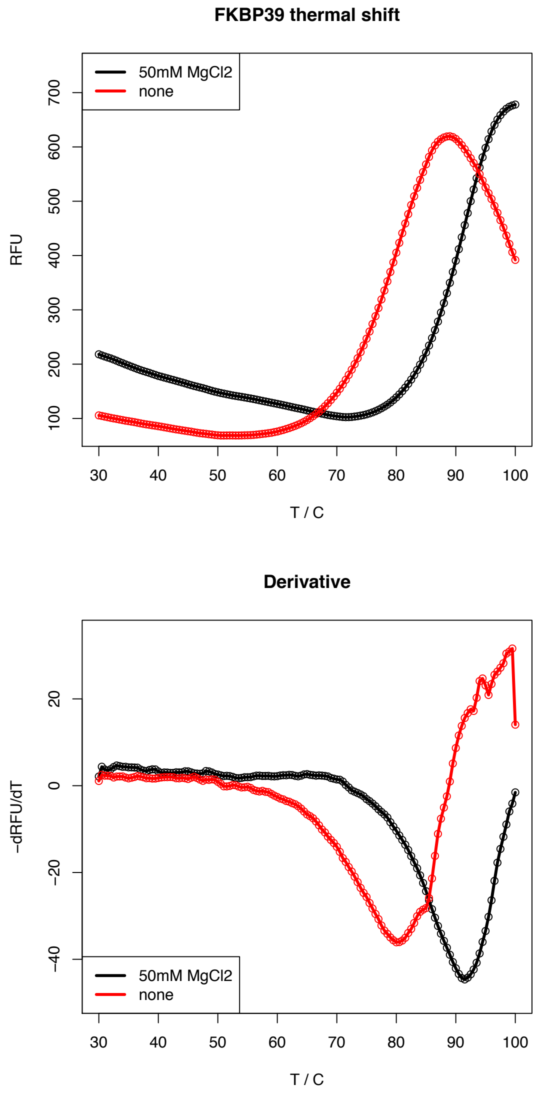

Supplementary Figure 1 Thermal shift assay of FKBP39 NPL-domain (residues 1-92). The two melting curves are FKBP39 in the presence or absence of 50 mM $MgCl_2$. The lower panel shows the temperature derivative to reveal the maximum. The two curves are separated by more than 10 °C.

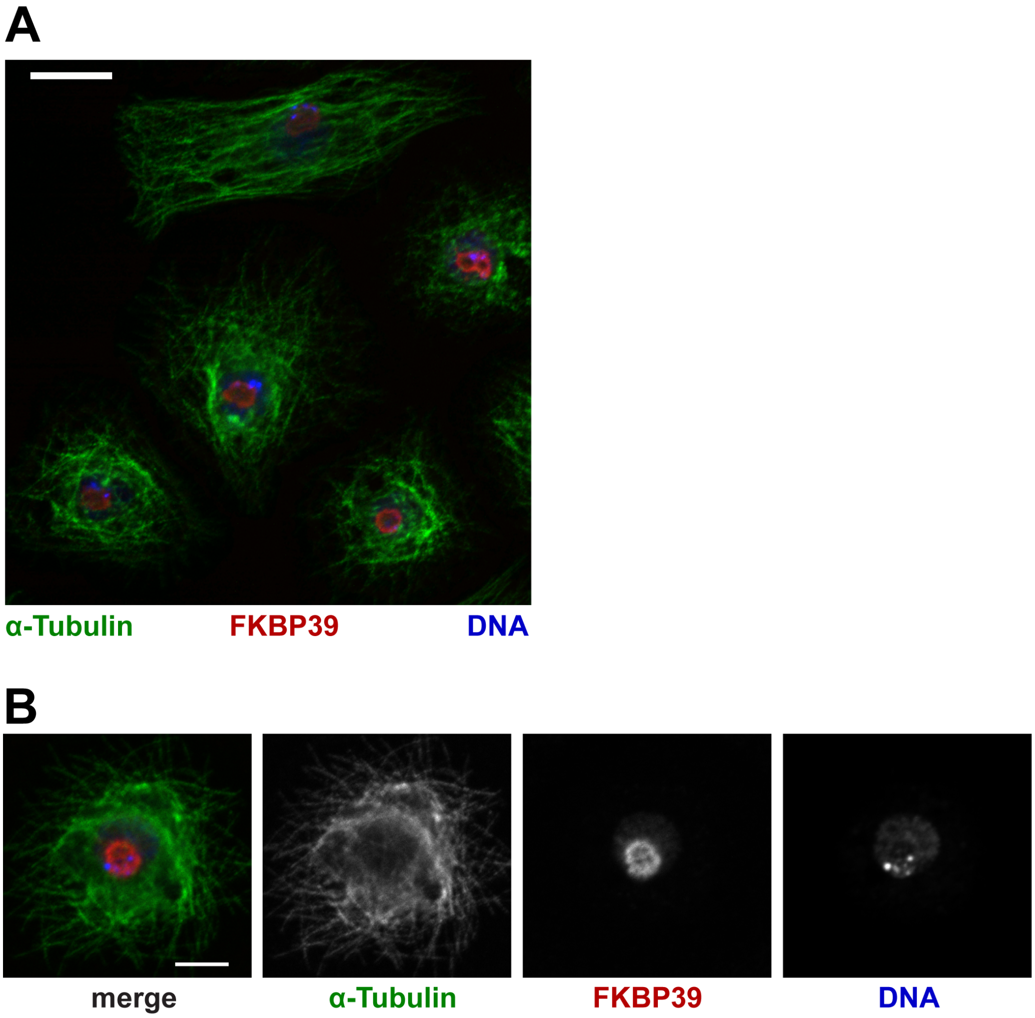

Supplementary Figure 2 FKBP39 localises to nucleoli in *Drosophila melanogaster* D.Mel-2 cells. (A) Field of view as seen in confocal microscope. Cultured D.Mel-2 cells were fixed and stained with specific antibodies to reveal the localisation of α-tubulin in green, FKBP39 in red and DNA in blue. *Drosophila* cells usually contain just one big nucleolus. Scale bar is 10 μm. (B) An exemplary interphase cell stained as in (A); images acquired in individual channels shown in grayscale. Scale bar is 5 μm.

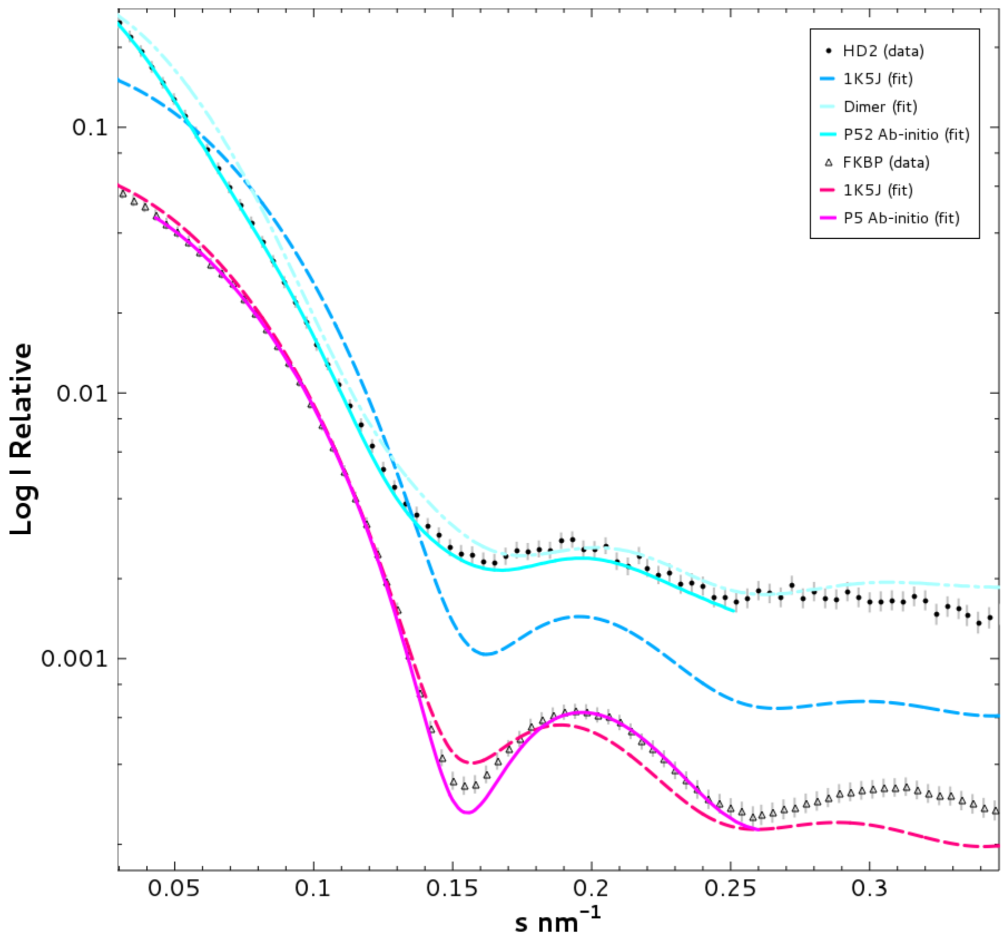

Supplementary Figure 3 SAXS scattering curves of FKBP39 and HD2 NPL. The figure shows experimental data for HD2 top (black circles) and FKBP39 bottom (triangles) overlaid with the fits of the *ab-initio* envelopes (solid lines) and structures (dashed) in shades of blue and pink for HD2 and FKBP respectively. HD2 has fits from the monomeric structure of nucleoplasmin (1K5J) and a putative dimer. Fits show a good agreement for a decameric configuration for HD2 and a pentameric configuration for FKBP39 in solution.

Supplementary Methods

Thermal shift

Buffers (50 mM final concentration) and salt (50-250 mM final concentration) were pipetted into a 96-well thin wall PCR plate (Bio-Rad, Hercules, CA). Protein (1 μM final concentration) and SYBRO orange (2x final concentration) were added and the plate sealed with optical film (Bio-Rad). The plates were placed into an iQ5 thermal cycler (Bio-Rad) and equilibrated at 25 °C for two minutes. Temperature was ramped from 25 to 95 °C in intervals of 0.5 °C and 30 seconds per interval.

Microscopy

Drosophila melanogaster D.mel-2 cells were cultured as described earlier. For the purpose of microscopy they were grown on Concanavalin A-coated cover slips and fixed with 4 % formaldehyde according to standard procedures. Staining and microscopy were performed exactly as in[30] using the following antibodies: mouse monoclonal anti-αTubulin (clone DM1α; SIGMA) at 1:1000, rabbit anti-FKBP39 (gift from Renato Paro, ETH) at 1:2000, and counter-stained with DAPI present in the Prolong Gold Anti-Fade mounting medium (Life Technologies). Images acquired on Zeiss Meta510 confocal microscope (100x objective) were processed using ImageJ (NIH, USA).
